# Coopetition Strategy in the Healthcare: Good or Bad?

**DOI:** 10.34172/ijhpm.8679

**Published:** 2024-12-14

**Authors:** Zahra Sadeqi-Arani, Esmaeil Mazroui Nasrabadi

**Affiliations:** ^1^Department of Business Management and Entrepreneurship, Faculty of Financial Science, Management and Entrepreneurship, University of Kashan, Kashan, Iran.; ^2^Department of Business Management, Faculty of Financial Science, Management and Entrepreneurship, University of Kashan, Kashan, Iran.

## Dear Editor,

 Today, the healthcare system faces three major challenges in delivering healthcare services: (*i*) shortages of medical equipment, hospitals, and qualified healthcare professionals^1,2^; (*ii*) growing overall demand due to a rising population and unforeseen events such as pandemics and natural disasters^3-5^; and (*iii*) escalating costs, which hinder affordability.^6^ Traditionally, these challenges are framed as the “Triple Aim” of healthcare: improving access, affordability, and quality.^7^ Addressing these interconnected issues is essential for creating a more resilient healthcare system. Additionally, the high costs of medical equipment and the need for knowledge-based services, which require skilled staff and significant investments, have driven healthcare organizations to collaborate with their competitors through coopetition.^5^ Coopetition is the collaboration between business competitors for mutual benefit.^8^ Coopetition arises when organizations encounter complex challenges that are difficult, to address independently^9^; particularly when these challenges also affect other entities within the same sector or geographic region. Therefore, competitors cooperate when they see aligned interests, aiming to create more value together than individually. By collaborating on shared obstacles, such as industry-wide regulatory requirements, resource constraints, or technological advancements, organizations can harness collective strengths to devise effective solutions.^10,11^ This approach enables them to address common issues while maintaining competitive differentiation in other areas. This value comes from a larger customer market, shared resources and knowledge, cost reductions, and cross-functional teams.^12^

 Coopetition in healthcare can take various forms and be applied in multiple areas. For example competing pharmaceutical companies can collaborate on the development of new drugs, particularly for complex diseases like cancer or rare genetic disorders. The collaboration between Pfizer and BioNTech to develop the COVID-19 vaccine exemplifies coopetition in healthcare. This partnership significantly contributed to the global fight against the pandemic.

 Healthcare organizations employ the coopetition strategy with several aims: achieving cost savings through resource sharing such as technology, equipment, and personnel^2,12^; enhancing innovation and research by sharing knowledge and conducting joint research^2,12,13^; expanding the market by increasing access to a larger patient base^14^; mitigating financial and operational risks associated with new technological projects and the risk of losing potential customers^15^; improving the quality and quantity of healthcare services provided^5^; enhancing patient care delivery through the sharing of knowledge, practices, care protocols, and an integrated approach to referrals and healthcare services^5,16^; and ultimately, strengthening the reputation and credibility of their brand.^17^

 Despite the numerous advantages of coopetition strategy, its implementation comes with some challenges and drawbacks. These include the risks of confidentiality and data security, as sharing sensitive patient information and key organizational data among partners can be problematic^18^; the risk of conflicts of interest and balancing competition and cooperation due to differing priorities and goals^19^; operational challenges in coordinating activities among various organizations and disputes over the allocation of shared resources^20^; legal and regulatory issues, such as ensuring that joint initiatives adhere to established health standards and protocols^21^; imbalances and inequalities in the benefits derived from coopetition, and the dependency of smaller institutions on larger organizations,^12,22^ which can create friction and reduce the effectiveness of collaboration. Intellectual property issues represent a significant barrier to coopetition, especially in sectors like healthcare where innovation and proprietary knowledge are critical. While intellectual property protection encourages innovation, it can create challenges in collaborative settings, as firms may be hesitant to share valuable information with competitors. This reluctance stems from concerns over losing competitive advantages and potential disputes over ownership and usage rights. In healthcare, such issues can delay innovation or limit collaboration.^23^ Addressing intellectual property challenges is crucial for enabling effective cooperation between competitors. Additionally, in some cases, coopetition may lead to collusion on pricing and access, which poses a threat to maintaining a competitive and accessible healthcare market, especially in regions where the number of healthcare providers is limited. This highlights the importance of balancing cooperation with regulatory oversight to protect market integrity.^24^ This potential downside underscores the importance of regulatory oversight to mitigate the risks of anticompetitive behaviors. Antitrust laws, such as the Sherman Act in the United States, are designed to prevent such practices and ensure that collaborations do not violate competition standards.^25^ Furthermore, competition authorities closely monitor healthcare collaborations to ensure that they do not stifle competition or harm consumer welfare. In some countries, regulations such as the “Sunshine Act” in the United States have been enacted to limit conflicts of interest and prevent undue influence in the healthcare sector.^26^

 Hence, it is crucial for healthcare organizations and institutions to meet specific prerequisites and requirements to fully leverage the advantages and minimize the disadvantages of coopetition. These prerequisites include:

- Utilizing flexible and dynamic business models and aligning strategies to adapt to the specific and changing needs and conditions of each organization.
- Implementing financing models to fairly distribute costs and revenues among collaborating organizations.
- Recruiting, retaining, and developing employees who are flexible, team-oriented, adaptable, creative problem-solvers, and continuous learners.
- Establishing clear agreements and contracts on ownership, licensing, and sharing of intellectual property that comply with national and international health regulations and protect intellectual property rights.
- Leveraging digital innovations such as telemedicine technologies, artificial intelligence, and data mining to share patient and management information, analyze health data, predict trends, and ensure data security and patient privacy through robust cybersecurity protocols.
- Building trust by clarifying benefits, managing conflicts of interest, and supporting smaller institutions in maintaining growth potential to eliminate dependency.
- Monitoring project progress and making key decisions through the formation of joint committees.
- Managing internal and external communications to ensure timely and accurate information exchange between units and to facilitate cooperation.
- Cultivating an organizational culture based on acceptance of change and collaboration through the active participation of all stakeholders in key decision-making processes.
- Continuously monitoring performance and project progress to identify and quickly rectify issues.
- Managing diversity to leverage various perspectives and experiences.

The extensive role of coopetition in healthcare has been widely recognized across various settings.^12^ Coopetition can drive major advancements and benefit society by combining the strengths of competing entities to achieve common goals. Organizations are advised to aim for an “optimal level” of coopetition since both insufficient and excessive coopetition can negatively impact different aspects of company performance.^22^ Adhering to these prerequisites is crucial for maximizing the advantages and minimizing the disadvantages of the coopetition strategy in the healthcare sector.

## Ethical issues

 Not applicable.

## Conflicts of interest

 Authors declare that they have no conflicts of interest.
